# Supplementary material for: Unveiling the Bioactive Potential of the Invasive Jellyfish Phyllorhiza punctata Through Integrative Transcriptomic and Proteomic Analyses
Source: Biomolecules. 2025 Aug 4;15(8):1121. doi: 10.3390/biom15081121 (PMC12383608; doi:10.3390/biom15081121)
Supplement: Supplementary file 1 [file biomolecules-15-01121-s001.zip › Suplementary Material/FileS3/PEPFOLD4/240_13873/clusters.html]

 PEP-FOLD clusters


Download archive of all models  here   

|  |  |  |
| --- | --- | --- |
| # | file | sOPEP |
| # cluster 1 - Size: 10 |  |  |
|  | 240-model1 | -15.685 |
|  | 240-model1.1 | -15.4907 |
|  | 240-model1.2 | -15.4907 |
|  | 240-model1.3 | -15.4677 |
|  | 240-model1.4 | -15.3455 |
|  | 240-model1.5 | -15.3455 |
|  | 240-model1.6 | -15.3455 |
|  | 240-model1.7 | -15.3298 |
|  | 240-model1.8 | -15.3298 |
|  | 240-model1.9 | -15.1649 |
| # cluster 2 - Size: 31 |  |  |
|  | 240-model2 | -15.6528 |
|  | 240-model2.1 | -15.5272 |
|  | 240-model2.2 | -15.4027 |
|  | 240-model2.3 | -15.4027 |
|  | 240-model2.4 | -15.4027 |
|  | 240-model2.5 | -15.3935 |
|  | 240-model2.6 | -15.3935 |
|  | 240-model2.7 | -15.3935 |
|  | 240-model2.8 | -15.3935 |
|  | 240-model2.9 | -15.3404 |
|  | 240-model2.10 | -15.3179 |
|  | 240-model2.11 | -15.3179 |
|  | 240-model2.12 | -15.3142 |
|  | 240-model2.13 | -15.3059 |
|  | 240-model2.14 | -15.304 |
|  | 240-model2.15 | -15.2773 |
|  | 240-model2.16 | -15.2773 |
|  | 240-model2.17 | -15.2773 |
|  | 240-model2.18 | -15.2368 |
|  | 240-model2.19 | -15.1565 |
|  | 240-model2.20 | -15.1565 |
|  | 240-model2.21 | -15.1565 |
|  | 240-model2.22 | -15.1565 |
|  | 240-model2.23 | -15.1565 |
|  | 240-model2.24 | -15.1565 |
|  | 240-model2.25 | -15.1444 |
|  | 240-model2.26 | -15.0981 |
|  | 240-model2.27 | -15.0925 |
|  | 240-model2.28 | -15.0763 |
|  | 240-model2.29 | -14.9245 |
|  | 240-model2.30 | -14.8225 |
| # cluster 3 - Size: 6 |  |  |
|  | 240-model3 | -15.3929 |
|  | 240-model3.1 | -15.3929 |
|  | 240-model3.2 | -15.3929 |
|  | 240-model3.3 | -15.1108 |
|  | 240-model3.4 | -14.7747 |
|  | 240-model3.5 | -14.5903 |
| # cluster 4 - Size: 20 |  |  |
|  | 240-model4 | -15.2981 |
|  | 240-model4.1 | -15.2981 |
|  | 240-model4.2 | -15.2981 |
|  | 240-model4.3 | -15.2981 |
|  | 240-model4.4 | -15.2981 |
|  | 240-model4.5 | -15.2981 |
|  | 240-model4.6 | -15.2981 |
|  | 240-model4.7 | -15.2981 |
|  | 240-model4.8 | -15.2981 |
|  | 240-model4.9 | -15.237 |
|  | 240-model4.10 | -15.1867 |
|  | 240-model4.11 | -15.183 |
|  | 240-model4.12 | -15.183 |
|  | 240-model4.13 | -15.1497 |
|  | 240-model4.14 | -15.1497 |
|  | 240-model4.15 | -15.1497 |
|  | 240-model4.16 | -15.0611 |
|  | 240-model4.17 | -15.0611 |
|  | 240-model4.18 | -14.9627 |
|  | 240-model4.19 | -14.7897 |
| # cluster 5 - Size: 3 |  |  |
|  | 240-model5 | -15.2555 |
|  | 240-model5.1 | -15.1438 |
|  | 240-model5.2 | -14.9496 |
| # cluster 6 - Size: 8 |  |  |
|  | 240-model6 | -15.2229 |
|  | 240-model6.1 | -15.2229 |
|  | 240-model6.2 | -15.0852 |
|  | 240-model6.3 | -14.9202 |
|  | 240-model6.4 | -14.9056 |
|  | 240-model6.5 | -14.7259 |
|  | 240-model6.6 | -14.6511 |
|  | 240-model6.7 | -14.178 |
| # cluster 7 - Size: 14 |  |  |
|  | 240-model7 | -15.1248 |
|  | 240-model7.1 | -15.1248 |
|  | 240-model7.2 | -15.1248 |
|  | 240-model7.3 | -15.1248 |
|  | 240-model7.4 | -15.1248 |
|  | 240-model7.5 | -15.1248 |
|  | 240-model7.6 | -15.1248 |
|  | 240-model7.7 | -15.1248 |
|  | 240-model7.8 | -15.0673 |
|  | 240-model7.9 | -15.0673 |
|  | 240-model7.10 | -15.0673 |
|  | 240-model7.11 | -15.0008 |
|  | 240-model7.12 | -15.0008 |
|  | 240-model7.13 | -14.7414 |
| # cluster 8 - Size: 3 |  |  |
|  | 240-model8 | -14.7546 |
|  | 240-model8.1 | -14.7082 |
|  | 240-model8.2 | -14.3084 |
| # cluster 9 - Size: 2 |  |  |
|  | 240-model9 | -14.7029 |
|  | 240-model9.1 | -14.5031 |
| # cluster 10 - Size: 2 |  |  |
|  | 240-model10 | -14.6934 |
|  | 240-model10.1 | -14.6592 |
| # cluster 11 - Size: 1 |  |  |
|  | 240-model11 | -14.2691 |
|  |  |  |
